# Ethnic groups’ knowledge, attitude and practices and Rift Valley fever exposure in Isiolo County of Kenya

**DOI:** 10.1371/journal.pntd.0005405

**Published:** 2017-03-08

**Authors:** Hippolyte Affognon, Peter Mburu, Osama Ahmed Hassan, Sarah Kingori, Clas Ahlm, Rosemary Sang, Magnus Evander

**Affiliations:** 1 International Crops Research Institute for the Semi-Arid Tropics (ICRISAT), Bamako, Mali; 2 International Centre of Insect Physiology and Ecology (ICIPE), Nairobi, Kenya; 3 Department of Clinical Microbiology, Virology, Umea University, Umea, Sweden; 4 Department of Clinical Microbiology, Infectious Diseases, Umea University, Umea, Sweden; Ministry of Health, KENYA

## Abstract

Rift Valley fever (RVF) is an emerging mosquito-borne viral hemorrhagic fever in Africa and the Arabian Peninsula, affecting humans and livestock. For spread of infectious diseases, including RVF, knowledge, attitude and practices play an important role, and the understanding of the influence of behavior is crucial to improve prevention and control efforts. The objective of the study was to assess RVF exposure, in a multiethnic region in Kenya known to experience RVF outbreaks, from the behavior perspective. We investigated how communities in Isiolo County, Kenya were affected, in relation to their knowledge, attitude and practices, by the RVF outbreak of 2006/2007. A cross-sectional study was conducted involving 698 households selected randomly from three different ethnic communities. Data were collected using a structured questionnaire regarding knowledge, attitudes and practices that could affect the spread of RVF. In addition, information was collected from the communities regarding the number of humans and livestock affected during the RVF outbreak. This study found that better knowledge about a specific disease does not always translate to better practices to avoid exposure to the disease. However, the high knowledge, attitude and practice score measured as a single index of the Maasai community may explain why they were less affected, compared to other investigated communities (Borana and Turkana), by RVF during the 2006/2007 outbreak. We conclude that RVF exposure in Isiolo County, Kenya during the outbreak was likely determined by the behavioral differences of different resident community groups. We then recommend that strategies to combat RVF should take into consideration behavioral differences among communities.

## Author summary

The Rift Valley fever (RVF) outbreak of 2006/2007 affected many regions in Kenya in varied degrees. The number of reported human cases and affected livestock herds varied between regions, but the reason for this variation has not been studied. We have investigated knowledge, attitude and practices differences between three ethnic communities in Isiolo County with different experiences of the 2006/2007 RVF outbreak. The pastoralist communities all had a relatively good knowledge regarding RVF, however the necessary preventive measures against RVF were not always practiced and the attitude towards RVF prevention were sometimes not good. When these results were analyzed on a community level, we found that the community with the best attributes had good preventive practices, positive attitude towards RVF prevention with less people and livestock affected by the disease. However, the combination of knowledge, attitude and practices was the determinant of avoiding RVF by the ethnic groups studied. These results indicate that understanding the behavior of the local communities could improve the preventive strategies to mitigate future RVF outbreaks.

## Introduction

Rift Valley fever (RVF) is a mosquito-borne viral disease which affects humans, livestock and other mammals [1, 2]. The disease is caused by Rift Valley fever virus (RVFV), an important hemorrhagic fever virus that occurs in Africa and the Arabic Peninsula [3, 4, 5]. Since 1930 when the first cases were diagnosed during an epizootic among sheep in the Rift Valley of Kenya, mitigation measures have often put emphasis on the veterinary and human disease perspectives. Often, the focus on managing RVF has been on monitoring and reporting of cases and death incidences to veterinary and public health authorities, managing human cases and deploying veterinary vaccines when available [6]. Although, the importance of knowledge, attitude and practices as drivers of infectious disease occurrence and spread among humans and animals has been established [7, 8], the contexts within which RVF occurs remain a nearly neglected research area [9, 10]. People behavior and practices play an important role in the spread of infectious diseases, and understanding the influence of behavior and practices on the spread of diseases can be crucial in improving prevention and control efforts [11]. Muga et al, [9] in a comprehensive literature review have shown that livestock sacrificial rituals, food preparation and consumption practices, gender roles are among the key factors that influence the transmission of RVF. Health outcomes are generally influenced by the social and cultural variables including socio-economic status (SES), such as educational level, income, and occupational status, ethnicity, gender, poverty and deprivation, in addition to aggregate characteristics of the social environments, such as the distribution of income, social cohesion and social capital etc. [12,13]. Ethnicity is a complex trait that is particularly useful and important because it possesses the social dimension necessary for understanding its impact on health outcomes. According to Shields et al, [14], ethnicity can be a powerful predictor for disease risk. The challenge is to demonstrate how health outcomes are influenced by many factors while recognizing that ethnicity and behavior differences may play an important role in their own right [15]. The objective of this study was to assess RVF exposure in Isiolo County in Kenya from the perspective of people behavior by comparing knowledge, attitudes and practices of three different communities (ethnic groups) and attempt to relate this to how they were affected by the RVF outbreak of 2006/2007.

## Methods

### Ethics statement

We sought and obtained the necessary approval to conduct the study from the Ethical Review Committee of the Kenya Medical Research Institute (KEMRI) (Non-SSC protocol No. 2346), to ensure adherence to Kenyan and international ethical guidelines (and regulations) governing research. We explained the purpose of the study to the research participants, local community and their leaders. During the data collection stage, all the respondents gave verbal consent. We ensured strict confidentiality in data handling and storage.

### Conceptual framework

To account for a set of attributes (socioeconomic status, levels of knowledge, attitude and practices) associated with the consequences of affecting exposure to RVF, the conceptual framework of multi-attribute utility theory is appropriate. The framework predicts: (i) behavior directly from an individual’s evaluation of consequences or (ii) outcomes associated with having or not having a given behavior [16]. The underlying assumption, having a given behavior such as refraining from RVF risk factors, would lead to a reduction in the number of infected people and animals. Therefore, the prediction of outcomes associated with having or not having a given behavior was considering two household health objectives regarding RVF: the reduction of the number of people and the number of animals affected by the disease. We hypothesized that the levels of the attributes may explain the health outcome of each household during the RVF outbreak of 2006/2007 in Kenya based on the behavioral differences assessed through the knowledge, attitude and practices among community groups.

### Study area and population

**Fig 1:**
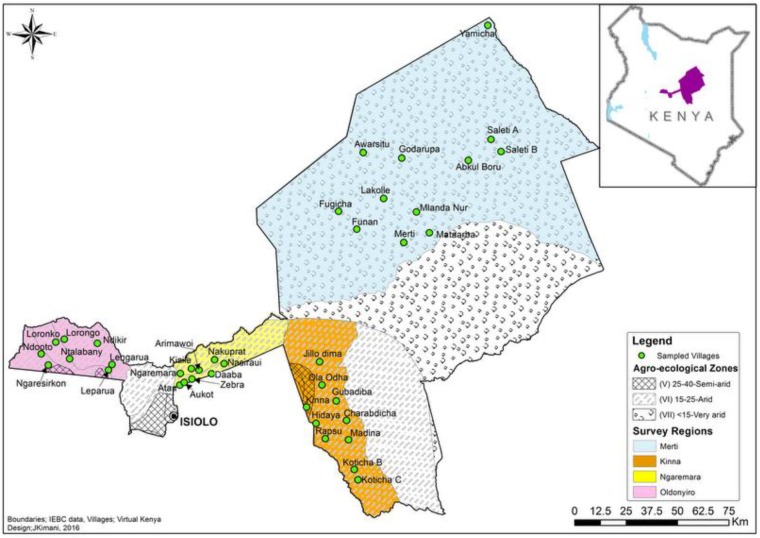
Map of Isiolo County with study area including sampled villages.

The study was conducted in Isiolo County, northern Kenya, one of the counties affected by the RVF outbreak of 2006/2007 and now classified as a medium risk county for RVF outbreak [17]. Isiolo County covers an area of 25,366 square kilometers between longitude 36°50’ and 39°30’ East and latitudes 0°5’ and 2° North (Fig 1). The county of Isiolo is characterized by the very arid, arid and semi-arid agro-ecological zones with an average annual rainfall of 350 mm and temperature of 29°C. The vegetation is comprised of shrubs and acacia trees, which supports rearing of camels, goats, sheep and cattle. Isiolo County is inhabited by five ethnic communities namely Maasai, Borana, Somali, Meru, and Turkana. According to the 2009 population and housing census, the county had a population of 143,294 people with a population density of about 6 people per square kilometers comprising of nomads and transhumants. In the present study, four divisions (Kinna, Merti, Ngaremara and Oldonyiro) were selected purposively due to the pastoralism practiced by residents. Kinna and Merti are inhabited mainly by the Borana ethnic group, Oldonyiro by the Maasai and Ngaremara by the Turkana. All three ethnic communities derive their livelihood from pastoralism; the Maasai and Borana are transhumants while the Turkana are nomads [18, 19].

### Study design and data collection

We conducted a cross-sectional study which allowed a single point data collection for each household head in the three communities in April 2014. A total of 698 households were selected randomly using sample frames provided by the veterinary officers of the locality. One hundred sixty (160) respondents were selected from the Maasai community, 175 from Turkana and 363 from Borana. The Borana community had the highest number of respondents because they are the majority in Isiolo County and occupy two of the selected divisions (Kinna and Merti). Household heads were interviewed with a structured questionnaire that had four sections which gathered various information such as household demography; knowledge about RVF disease in humans and animals, attitude and perception, and practices that would promote or prevent spread of the disease. Also, information was collected from the communities about the number of people and animals that were affected during the RVF outbreak of 2006/2007 as mentioned by the interviewees. Data were entered in Microsoft Excel Sheet and later cleaned and transferred to STATA software version 13 (StataCorp LP, TX, USA) for analysis.

### Data analysis

One-way ANOVA was used to compare ethnic groups’ characteristics and means were separated using Bonferroni adjustment at 95% confidence interval [20]. Spearman correlation test was used for the correlation between two variables. Composite indices were computed for knowledge, attitude and practice (KAP) for each household head. Generally, information collected in social science to describe perceptions and attitudes of people involves the use of scales either binary or multiple. We aimed at quantifying constructs such as KAP which are not directly measurable, by using multiple-item scales and summated weighted ratings to quantify the constructs of interest. Principal Component Analysis (PCA) was used to generate the composite indices for Knowledge score, Attitude score, Practice score and a composite of the three denoted KAP-score [21]. Thirty-five knowledge questions, eight attitude and nine practice questions (see supplementary document) were used in the computation for the indices. Answers to the questions were coded 1 for correct and 0 for incorrect based on established risk factors that are known to facilitate the spread of RVF [9, 22, 23]. The communities are pastoralists and their main livelihood is livestock keeping. The number of animals owned was measured through tropical livestock unit (TLU). TLU allows for computation of different livestock types into one standard measure where one TLU is equivalent to an animal of 250 kg live weight [24]. In addition, computation of a household dependency ratio which expresses the economic burden on the working age population, involved computation of dependents of the percentage of the working-age population [25].

## Results

### Household socio-economic characteristics

Most of the household heads interviewed in the three ethnic groups (Maasai, Borana and Turkana) were male with the Turkana community having the largest number (40%) of female headed households (Table 1). The average age of the household head was 45.5±0.5 years, however, those from the Borana community were significantly older (p < 0.01) compared to their counterparts of Turkana and Maasai communities. The average years of formal education of the household heads among the three communities was 3.1 with the least and most educated household head having 0 and 20 years of schooling respectively. The average number of people living in the household was 7.17±0.12 with the Turkana and Maasai having significantly more (p < 0.01) persons per household compared to the Borana community (Table 1). A comparison of the economic dependants showed that the average dependency ratio for the three communities was 142.6% with the Maasai and Turkana having significantly more (p < 0.01) dependants than the Borana community. The mean TLU for the three communities was 29.2 units with the Maasai having significantly more (p < 0.01) animals than the Borana and Turkana communities. In terms of the religion, results show that the majority (97.5%) of the Borana are Muslim, while the majority of the Turkana (98.3%) and the Maasai (98.75%) are Christian.

**Table 1:** Household characteristics of the study participants by community group.

Household Characteristic	Community groups		
	Turkana (n = 175)	Borana (n = 363)	Maasai (n = 160)
Gender (% of male)	60±4**a**	80±2**c**	70±4**b**
Gender (% of female)	40±4**a**	20±2**c**	30±4**b**
Age of household head (years)	43.5±1.2**a**	47.7±0.7**b**	42.5±1.1**a**
Years of schooling of the household head	0.8±0.1**a**	4.1±0.2**b**	3.2±0.3**b**
Number of household members	7.5±0.2**b**	6.6±0.1**a**	8.1±0.3**b**
Dependency ratio (%)	153.2±8.4**b**	127.6±5.8**a**	165±9.2**b**
Tropical Livestock Units (TLU)	15.5±1.7**a**	28.3±1.4**b**	46.4±3.8**c**

**Note**: Data in the table shows the means or percentage (%) and their standards errors. Means in the same row, followed by same letters are not significantly different at 5%.

### Key risky behavior encouraging spread of RVF disease

Table 2 presents a summary of the risky behavior observed in the three community groups.

**Table 2:** Risky behavior of the Maasai, the Borana and the Turkana in relation to RVF.

Variable description	Community groups (%)		
	Turkana (n = 175)	Borana (n = 363)	Maasai (n = 160)
**Handling of sick animals**			
Separate and bring to veterinary clinic	0	1.38	1.25
Left the animals to graze with the rest and when separated they were treated the sick animals themselves at home	100	98.62	98.75
**Handling of dead animals**			
Burn and bury	1.71	2.48	4.38
Consume the animal or throw outside to be eaten by dogs	98.29	97.52	95.63
**Slaughter animals for meat inside the home**			
Yes always	94.86	95.04	100
No	5.14	4.96	0
**Dispose carcass waste after slaughtering at home**			
Burn and bury	0.57	9.09	0.63
Consume carcasses or throw outside to be eaten by dogs	99.43	90.91	99.38
**Drink un-boiled milk**			
Yes	94.86	88.71	95.63
No	5.14	11.29	4.38
**Help animals to deliver**			
Yes	99.43	96.42	98.75
No	0.57	3.58	1.25
**Buy meat slaughtered from a slaughter house and checked by a veterinary officer**			
Yes	86.86	39.94	69.38
No	13.14	60.06	30.63
**Use mosquito bed nets**			
Yes	28.00	75.48	87.50
No	72.00	24.52	12.50

#### Sheltering and handling sick and aborted animals

Sheltering of animals (cattle, sheep, goats, camels and donkeys) inside the homestead was practiced by 96% of the Maasai. However, the Borana (89%) and the Turkana (51%) sheltered their animals outside and away from their homesteads. Results show that handling of sick animals, aborted fetus and helping aborted animals with bare hands (without gloves or protection), was practiced by 85% of the Turkana, 82% of the Maasai and 72% of the Borana. The comparison of the community groups indicates that the Maasai and the Turkana were significantly equally exposed while the Borana were statistically significantly less exposed compared to the Maasai and the Turkana (p < 0.05).

#### Veterinary inspection of slaughtered animals, consumption of uncooked meat and dead animals

The inspection of slaughtered animals by the veterinary office was less practiced by the three community groups. However, the Borana (29%) slaughtered statistically significantly (p < 0.05) more animals under veterinary supervision followed by the Maasai (19%) and the Turkana (6%). The majority (59%) of the Turkana consumed raw meat significantly more (p < 0.01) compared to the Borana (41%) followed by the Maasai (38%). However, no statistical difference could be observed between the Borana and the Maasai.

### Knowledge, attitude and practices scores about RVF

The average RVF knowledge score among the three community groups was 65.2±0.6 units (Table 3). However, the Maasai had significantly more knowledge (p < 0.01) about RVF compared to the Borana and the Turkana (Table 3). The attitude score that was comprised of the pastoralist’s perspective towards the sick animals, and people sick with RVF, showed that the Borana had a significantly higher (p < 0.01) attitude score compared to the Turkana and the Maasai. The practice score that was comprised of the recommended practices safeguarding a household from RVF, was statistically higher for the Borana compared to the Turkana. However, no significant difference could be observed between the Borana and the Maasai, while the difference was significant between the Maasai and the Turkana (Table 3). The KAP score of the Maasai was significantly higher (p < 0.01) compared to that of the Borana and the Turkana but no significant difference could be observed between the Borana and the Turkana.

**Table 3:** Community groups knowledge, attitude and practice scores.

	Community groups (Average score)			
Category of score*	Overall mean	Turkana (n = 175)	Borana (n = 363)	Maasai (n = 160)
Knowledge score	65.2±0.6	63.7±1.05**a**	62.7±0.8**a**	72.6±1.3**b**
Attitude score	67.6±0.7	59.1±1.2**a**	76.9±0.7**b**	55.7±1.4**a**
Practice score	14.1±0.3	12.4±0.3**a**	14.9±0.5**b**	14.0±0.7**b**
KAP score	68.4±0.5	65.3±1.09**a**	67.7±0.8**a**	73.4±1.1**b**

**Note:** Data in the table shows the average score (%) and their standards errors. Means in the same row, followed by same letters are not significantly different at 5%.

* the value of the scores (%) range from 0 to 100.

### RVF burden measured as the number of animals and people affected during the 2006/2007 outbreak

People and livestock affected by RVF, according to the communities, was used as proxy for the burden of the disease. The Borana and the Maasai communities suffered relatively less compared to the Turkana in terms of the proportion of livestock (per 1,000 livestock heads) affected by RVF during the 2006/2007 outbreak. However, results showed that there was no significant difference between the Borana and the Maasai (Table 4). The proportion of persons (per 1,000 people) affected by the disease during the 2006/2007 outbreak among the three community groups was not statistically significantly different, though the Turkana indicated the highest proportion of people affected followed by the Borana and the Maasai in the decreasing order (Table 4).

**Table 4:** Proportion of people and livestock affected by RVF as indicated by the community groups during the 2006/2007 outbreak.

	Community groups		
	Turkana (n = 175)	Borana (n = 363)	Maasai (n = 160)
Number of livestock affected by RVF per 1,000	281.36±62.06**b**	76.55±12.75**a**	81.87±33.12**a**
Number of persons affected by RVF per 1,000	25.06±5.78**a**	18.81±8.58**a**	14.14±4.24**a**

**Note**: Data in the table shows the mean number of livestock (cattle, sheep, goats, camels and donkeys) and the number of persons affected and their standards errors. The number of livestock and persons were derived for each household and extrapolated for 1,000 of people or animals. Means in the same row, followed by same letters are not significantly different at 5%.

### Association between RVF burden and community groups’ Knowledge, attitude and practices

The knowledge score (knowledge about RVF) of the respondents had significant and negative association with the number of animal affected in the Turkana (p < 0.05) and the Maasai (p < 0.1) groups (Table 5) meaning the less the community has knowledge about RVF, the more animal are affected during the outbreak. However, though the association was negative with the Borana, it was not statistically significant. For the number of people affected there was no significant difference between the Borana and the Maasai (Table 4); however, the association between the knowledge score and the number of people affected was positive and significant for Borana and negative and non-significant for the Maasai (Table 5).

**Table 5:** Association between RVF burden and community groups’ Knowledge, attitude and practices.

	Community groups					
	Turkana (n = 175)		Borana (n = 363)		Maasai (n = 160)	
	Spearman's rho	*p*-value	Spearman's rho	*p*-value	Spearman's rho	*p*-value
**Knowledge score**						
Number of animal affected by RVF	-0.232	0.002	-0.045	0.397	-0.142	0.074
Number of people affected by RVF	-0.090	0.237	0.123	0.020	-0.076	0.339
**Attitude score**						
Number of animal affected by RVF	-0.145	0.056	0.043	0.415	0.002	0.981
Number of people affected by RVF	-0.156	0.040	0.002	0.972	-0.188	0.017
**Practice score**						
Number of animal affected by RVF	0.113	0.137	0.008	0.881	0.031	0.695
Number of people affected by RVF	-0.035	0.650	0.102	0.052	-0.052	0.512
**Overall KAP score**						
Number of animal affected by RVF	-0.259	0.001	0.030	0.567	-0.069	0.385
Number of people affected by RVF	-0.196	0.009	0.022	0.677	-0.059	0.459

Note: Spearman's rho assesses how the relationship between two variables can be described.

The attitude score of the Turkana has a negative and significant correlation with the number of animals and the number of people affected (Table 5). This community group was the most affected compared to the Borana and Maasai (Table 4). For the Borana the association between the attitude score and the number of animals and the number of people affected was positive but not statistically significant. For the Maasai, the association between the attitude score and the number of animals affected was positive but not statistically significant. However, the association between the attitude score and the number of people affected was negative and significant (p < 0.05). The Maasai are more knowledgeable (Table 3) and their attitude score was also negatively associated with the number of people affected; they were less likely at risk as demonstrated by the number of animals and people affected in this community (Table 4).

The association between the practice score and the number of the animals affected during the outbreak was positive but not statistically significant for the three community groups meaning that they are all vulnerable to RVF in terms of risky practices. For the number of people affected the association was negative for Turkana and the Maasai and positive but not statistically significant and also positive for the Borana but only significant at 10% (Table 4).

Results of the study revealed a negative and significant association between total KAP score and the number of animals and people affected among the Turkana who suffered more during the outbreak compared to the Borana and the Maasai. The Turkana had less KAP score (Table 3) and this may explain why they were more affected in terms of the number of sick animals and people affected compared to the Borana and the Maasai (Table 4). The Maasai had significantly more KAP score (Table 3) and were less affected. However, for this less affected community, the correlation between total KAP score and the number of animals and people affected among Maasai was negative but not statistically significant.

## Discussion

The importance of behavioral factors as determinants of RVF disease occurrence and spread has previously been documented [7, 9, 26, 27, 28]. However, little is known on the effect of specific ethnic groups’ behavior on RVF exposure. Results from this study indicated that the Turkana were more affected followed by the Borana and the Maasai in terms of the proportion of people in the household affected by RVF during the 2006/2007 outbreak, but the difference between the three community groups was not statistically significant. A single death in a household can be devastating for a family so the occurrence and the loss of persons, one or many, is what matters. However, as far as the number of animals affected is concerned, the Maasai and the Borana were less impacted in terms of the proportion of livestock affected by RVF. This can be explained by the fact that Maasai and the Borana were significantly more educated compared to the Turkana, as measured by the average number of years of schooling of the household heads. An association between higher educational attainment and better health status has been repeatedly reported in literature. Kawachi et al, [29] have demonstrated that there is evidence to suggest that schooling is causally related to improvements in health outcomes. However, they pointed out that much remains to be known for example, what type of education matters for health. Schooling helps people choose healthier life-styles by improving their knowledge of the relationships between health behaviors and health outcomes [30]. At the time of the study, the three community groups had reasonable knowledge about RVF as expressed by their knowledge score (Table 3). However, the Maasai that have significantly higher knowledge score were the least affected in terms of the proportion of livestock sick of the RVF disease.

The results of the study have shown that religion did not influence the burden of RVF outbreak on the affected population, although the majority of the Maasai and the Turkana are Catholic, they were affected differently by the disease. However, a study by Gray demonstrated that among 38 sub-Saharan African countries, the percentage of Muslims within countries negatively predicted HIV prevalence [31]. Also, every year, because of religion, millions of small ruminants are slaughtered during the religious festivals at Mecca. The risk of infection is high at the time of slaughtering, when aerosols of infected blood may be generated, particularly by traditional sacrificial slaughtering practices [32]. This sacrificial slaughtering may represent a risk of infection, but no RVF disease incident has been reported. However, it was then strongly recommended by the author that the movement of sheep and goats to Mecca for the religious festivals should be strictly prohibited from any area in which epizootic RVF virus has occurred in the previous three to six months [32].

Close contact and handling of sick animals is a potential risk factor for contracting RVF. More people in the Borana community group applied good practices, such as wearing gloves when handling sick animals, taking care of aborted fetuses, and helping animals to deliver as compared to the Turkana who were more affected by RVF. Many emerging diseases are zoonotic infectious diseases transmitted between animals and humans; examples include RVF [26, 33, 34, 35]. Applying good practices by wearing protective gloves can significantly reduce the risk of being infected through transmission of the disease between animals and humans. It has been shown in China that the interaction of people with animals favors the emergence and the spread of new microbial threats [36].

The proportion of female headed households was significantly higher in the Turkana community compared to the Borana and the Maasai. In sub-Saharan Africa, women frequently spend more time than men in animal care [10, 37]. This may explain why Turkana were more affected in terms of the number of people sick of the disease during the outbreak. However, it is not clear how gender differentiation influences the spread of RVF [10, 37].

Responsibilities of veterinary services include epidemiological surveillance of animal diseases and ensuring the safety and suitability of meat for consumption [38, 39, 40]. The Borana community group who slaughtered more animals under the veterinary inspection was the less affected group in terms of the proportion of animals sick of RVF during the 2006/2007 outbreak, compared to the Turkana and the Maasai, although the difference between the later was not statistically significant. Also, the proportion of people in the household sick of RVF was less in the Borana community group compared to the Turkana, though the difference was not statistically significant. The objectives of meat inspection are twofold; first, meat inspection ensures that only healthy physiologically normal animals are slaughtered for human consumption and secondly is to ensure that meat from animals is free from disease and represent no risk to human health [38, 39, 40].

This study showed that the Maasai had a higher KAP score compared to the Boran and the Turkana who were more affected in terms of the proportion of livestock sick of RVF. Though the attitude score was not statistically significantly different between the Maasai and the Turkana, they were differently affected by the disease. This can be explained by the fact that the Maasai had significantly higher knowledge score compared to the Turkana. Also, the Maasai had significantly higher practice score compared to the Turkana. The Borana community group was not different from the Maasai in terms of practice score, though the Maasai were more knowledgeable than them. Moreover, the Borana community group had significantly better attitude compared to the Maasai as shown by their higher attitude score (Table 3). This study showed that to better describe what is important for the disease burden of the affected community is the combination of knowledge, attitude and practices. Generally, higher knowledge score about a specific disease is not always translated into better practices. For instance, farmers’ practical ability to diagnose African animal trypanosomiasis was higher than suggested by their knowledge about the disease [41]. The high KAP score of the Maasai may explain why they were less affected by the RVF disease during the 2006/2007 outbreak. Behavioral differences were important in explaining why various communities were affected differently by the RVF outbreak in Isiolo County, Kenya in 2006/2007.

## Conclusion

Better knowledge about a specific disease is not always translated into appropriate practices, but rather the application of good practices together with the right attitude, as applied by one of the communities in our study, may explain why this community was less affected by RVF disease. Also, the combination of people´s knowledge, attitude and practices in a single index is more appropriate to explain disease burden rather than the single element taken separately. We conclude that RVF exposure in Isiolo County, Kenya during the RVF outbreak, was to a large extent determined by the behavioral differences of different community groups. Therefore, we recommend that strategies to combat RVF should take into consideration sociocultural and behavioral differences among communities.

## Supporting information

S1 Checklist STROBE Checklist. (DOC)

S1 Dataset Dataset of items included in reports of cross-sectional studies. (XLSX)

S1 Table Knowledge, Attitude and Practices Questions. (DOCX)
