# Supplementary material for: Ethnic groups’ knowledge, attitude and practices and Rift Valley fever exposure in Isiolo County of Kenya
Source: PLoS Negl Trop Dis. 2017 Mar 8;11(3):e0005405. doi: 10.1371/journal.pntd.0005405 (PMC5358895; doi:10.1371/journal.pntd.0005405)
Supplement: S1 Table — (DOCX) [file pntd.0005405.s003.docx]

Knowledge Attitude Practices Questions

| **RVF KNOWLEDGE ASSESSMENT QUESTIONS** | |
| --- | --- |
| K_1 | What is the RVF host range? |
| K_2 | Is RVF contagious from person to person? |
| K_3 | Do you avoid eating uncooked meat? |
| K_4 | Do you slaughtering animals under veterinary supervision? |
| K_5 | Do you avoid handling sick or aborted animal? |
| K_6 | Who is the most suitable person to diagnose RVF? |
| K_7 | Do human patients need medical tests for RVF? |
| K_8 | Is human blood sample the best for RVF test? |
| K_9 | Who is the most susceptible person to RVF? |
| K_10 | Which is the most affected human age group by RVF? |
| K_11 | Who is the most affected workers by RVF? |
| K_12 | Which is the most affected animal by RVF? |
| K_13 | Which is the most affected animal age group by RVF? |
| K_14 | Do animal get infected by direct contact? |
| K_15 | Can RVF be spread among animals through infected mosquito bites? |
| K_16 | Can RVF be prevented through vaccination? |
| K_17 | Can RVF be prevented by isolating sick animal? |
| K_18 | Can we prevent RVF infection by slaughtering animals under veterinary supervision? |
| K_19 | Can we prevent RVF by isolating aborted animals? |
| K_20 | Who is the most suitable person to diagnose RVF in animals? |
| K_21 | Do animals sick with RVF need medical tests? |
| K_22 | Is a blood sample test necessary for animals to verify RVF? |
| K_23 | Is RVF spread in certain season of the year? |
| K_24 | Which season is RVF more common? |
| K_25 | Did noticed any changes in rain during the2007 RVF outbreak? |
| K_26 | Have you heard that RVF can spread to other country parts? |
| K_27 | Can RVF spread from one country to another? |
| K_28 | Can RVF disease lead to animal trade ban between regions in the country? |
| K_29 | Can RVF lead to animal trade ban outside the country? |
| K_30 | Can a person catch RVF through direct contact with an infected animal? |
| K_31 | Can a person get RVF through direct contact with an infected mosquito bite? |
| K_32 | Can a person get RVF through eating non-cooked meat? |
| K_33 | Can a person get RVF through taking non-boiled milk? |
| K_34 | Is Mosquito an animal RVF vector? |
| K_35 | Can RVF spread from one region to another with a country? |

| **RVF ATTITUDE ASSESSMENT QUESTIONS** | |
| --- | --- |
| A_1 | Should an RVF patient avoid certain foods? |
| A_2 | How do you treat an RVF patient? |
| A_3 | Is there any need to isolate an RVF patient? |
| A_4 | Is there need to quarantine an animal with RVF? |
| A_5 | Are agricultural areas more prone to RVF than cities? |
| A_7 | What is the role of the community in preventing RVF? |
| A_8 | Can you get RVF if it spread in your area? |
| A_9 | Why do you think you can get? |
|  |  |
| **RVF PRACTICE ASSESSMENT QUESTIONS** | |
| P_1 | How do you handle sick animals? |
| P_2 | How do you handle aborted animals? |
| P_3 | What do you do with dead animals |
| P_4 | Do you slaughter animals for meat inside the homestead? |
| P_5 | How do you dispose carcass waste when you slaughter animals for meat at home? |
| P_6 | Do you drink un-boiled milk? |
| P_7 | Do you help animals to deliver at home? |
| P_8 | Do you buy meat slaughtered from a slaughter house and checked by a veterinary officer? |
| P_9 | Do you use a mosquito bed net? |
